# Negligible Impact of Drought-Resistant Genetically Modified Maize on Arthropod Community Structure Observed in a 2-Year Field Investigation

**DOI:** 10.3390/plants11081092

**Published:** 2022-04-18

**Authors:** Jun-Qi Yin, Da-Ming Wang, Jin-Gang Liang, Xin-Yuan Song

**Affiliations:** 1 Agro-Biotechnology Research Institute, Jilin Academy of Agricultural Sciences, Changchun 130033, China; yin_junqi@163.com (J.-Q.Y.); jumina054824@163.com (D.-M.W.); 2 Development Center of Science and Technology, Ministry of Agriculture and Rural Affairs, Beijing 100176, China

**Keywords:** drought-resistant genetically modified maize, arthropods, abundance, diversity, community composition

## Abstract

Dehydration-responsive element-binding (DREB) transcription factors regulate diverse processes during plant development. Here, a 2-year field study was conducted to assess the potential effects of DREB-genetically modified maize (GM1) on arthropod species and ecological communities. Arthropod abundance, diversity, and community composition in GM1 and its non-transformed counterpart maize variety, Chang 7-2, were compared using whole plant inspection, pitfall trap, and suction sampler methods. Based on Shannon–Wiener diversity, Simpson’s diversity, Pielou’s indexes, number of species, and total number of individuals, GM1 had a negligible effect on arthropod abundance and diversity. Redundancy analysis indicated that the composition of arthropod community was not associated with maize type in the three investigation methods, while it exhibited significant correlation with year and sampling time in whole plant inspection and suction sample methods, and distinctly correlated with sampling time in the pitfall trap method. Nonmetric multidimensional scaling analysis of variable factors in the three investigation methods showed that sampling time, rather than maize type or year, was closely related to the composition of arthropod community in the field. Our results provide direct evidence to support that DREB-GM maize had negligible effects on arthropods in the Jilin Province under natural conditions.

## 1. Introduction

Worldwide, adverse environmental factors can cause up to 70% loss of crop yields, with drought being the most important abiotic stress factor affecting maize production. Furthermore, global warming has intensified the frequency of occurrence of drought, which, in recent years, has affected 2 × 10^7^ hm^2^ annually in China, severely reducing maize yields [1,2,3].

With the development of genetically modified crops, the safety of transgenic plants and their impact on biodiversity have become increasingly important for research [4]. In China, 10 plant varieties have been approved for agricultural GM safety certificates, including insect-resistant and herbicide-resistant maize. Drought-resistant genetically modified maize has great potential to alleviate conditions of drought, and it is currently being actively researched and developed. Additionally, the arthropod community is important for maize production because the species involved play a crucial role in maintaining the ecological balance in farmlands [5,6,7,8,9,10,11,12]. Drought-resistant genetically modified crops show drought resistance mechanisms that improve their water use efficiency (WUE); however, whether the genetic modifications may have an impact on arthropods is unknown [13], although there are few reports on the impact of drought-resistant crops, especially on arthropods. Thus, China’s agricultural genetically modified organisms (GMO) safety management measures require that genetically modified maize must be subjected to a rigorous environmental safety assessment before commercialization, among which the impact assessment on the arthropod community is an important part [14,15]. To date, many laboratories have conducted field trials to study the potential ecological effects of genetically modified crops on non-target organisms, while most of them have focused on insect-resistant or herbicide-resistant crops [16,17,18,19,20,21]. At present, most studies on genetically modified drought-resistant crops have focused on gene discovery and functional verification, while environmental safety assessments are limited to the evaluation of competitiveness for survival and its effects on available nutrients, enzyme activities, and microbial community diversity in rhizosphere soil [22,23,24,25,26,27,28,29]. However, the effects of drought-resistant genetically modified maize on farmland ecosystems have rarely been reported. Thus, whether drought-resistant maize harms arthropods in the field remains unknown.

The safety assessment of genetically modified maize needs to be conducted for a long-term study. Herein, we used three methods to evaluate the effects of GM1 on arthropods in 2016 and 2017 in a field study that aimed to evaluate whether genetically modified drought-resistant maize, GM1, had a significant impact on the diversity and composition of arthropods. Although many field and laboratory studies have confirmed that the effects of GM crops on arthropods were not significant [30], their influence needs to be analyzed case-by-case as, although some may have no influence on the overall ecological function, they do alter the composition of arthropods and dominant species [4]. This comprehensive study will provide useful data for the commercialization of GMO in the future.

## 2. Results

### 2.1. Arthropods in Transgenic and Non-Transgenic Maize Plots

**Figure 1:**
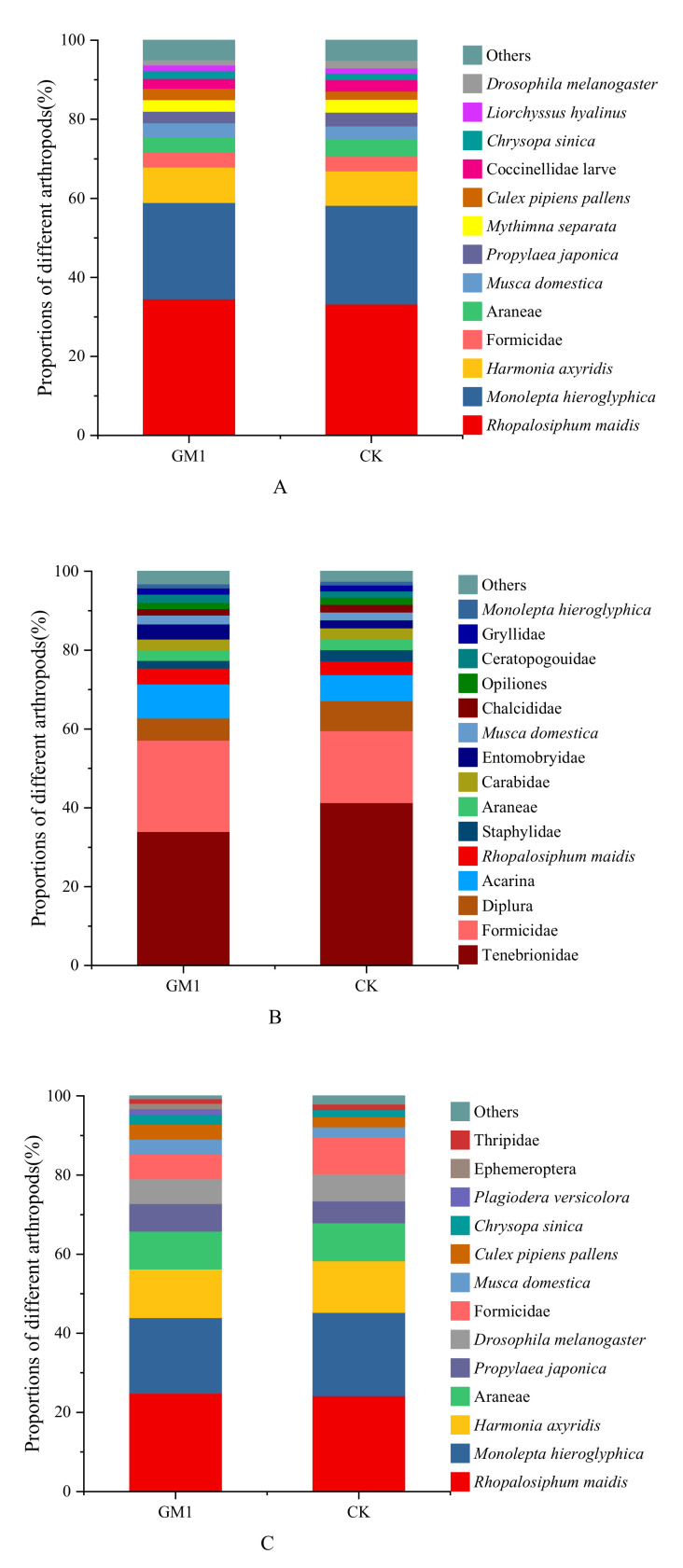
Proportions of arthropods found in GM1 and CK maize plots in 2016 and 2017 by (**A**) whole plant inspection, (**B**) pitfall trap, and (**C**) suction sampler. The taxa with a proportion greater than 1% are shown individually, while taxa with a proportion smaller than 1% are shown together (others).

In this study, arthropods that were easy to classify were identified to species level, such as *Rhopalosiphum maidis* and *Monolepta hieroglyphica*. Arthropods which were difficult to classify were identified to family level, such as Tenebrionidae and Formicidae. Through whole plant inspection, 2249 arthropods of 32 species and families in transgenic and 2130 of 33 species and families in non-transgenic maize (CK) were recorded over the 2-year experimental period. *R. maidis* and *M. hieroglyphica* accounted for 34.59% and 24.37%, and 33.24% and 24.98%, in transgenic and non-transgenic maize fields, respectively (Figure 1A). In turn, using the pitfall trap method, we recorded 3585 arthropods of 32 species and families, and 3650 arthropods of 33 species and families, in transgenic and non-transgenic maize, respectively, over 2 years. The occurrences of arthropods in the two types of maize were also similar in terms of taxon and number over the 2 years. Furthermore, in the two types of maize field, Tenebrionidae and Formicidae were the dominant families, accounting for 34.00% and 23.12%, and 41.34% and 18.22%, in transgenic and non-transgenic maize, respectively (Figure 1B). Finally, using the suction sampler method, a smaller number of arthropods was recorded, with a total of 430 arthropods of 16 species and families recorded in transgenic maize and 437 arthropods of 16 species and families in non-transgenic maize, over the 2 years of study. *R. maidis*, *M. hieroglyphica*, and *Harmonia axyridis* were the dominant taxa, accounting for 24.88%, 19.07%, and 12.33%, and 24.26%, 21.05%, and 13.04% in transgenic and non-transgenic maize fields, respectively (Figure 1C). Further, we analyzed the difference of proportion of *R. maidis*, which all occurred largely in the three methods, and the results show that there was no significant difference in the proportion of *R. maidis* (*p* > 0.05).

### 2.2. Impacts of Maize Type, Year, and Sampling Time on Abundance and Diversity of Arthropods

We sampled arthropods using the whole plant inspection, pitfall trap, and suction sampler methods. The effects of maize type, year, sampling time, and their interactions on arthropod abundance and diversity indices were evaluated using Shannon–Wiener index (*H′*), Simpson’s diversity index (*D*), number of species (*S*), Pielou’s evenness index (*J*), and the total number of individuals (*N*). Unequally spaced repeated-measures ANOVA results showed that maize type had no significant effect on *H′*, *D*, *J*, *S*, or *N* of arthropods, while sampling time did have a significant impact on all these parameters; however, the interactions between maize type and year, maize type and sampling time, and between maize type and year and sampling time did not influence any of them, regardless of sampling method. Furthermore, ANOVA showed that year had a significant impact on *H′*, *D*, and *J*, when investigated using whole plant inspection or pitfall trap, but only year influenced *D* and *J* significantly when the suction sampler method was used. The above results indicate that arthropods were affected by sampling time but not by maize type. Meanwhile, two critical arthropod groups with different feeding habits (*Rhopalosiphum maidis* feed on maize, Formicidae do not eat maize directly) were selected to analyze the difference of their abundance in order to verify the impact of maize types on biodiversity of critical groups, and the results show that the differences were not significant (*p* > 0.05). Specifically, genetically modified maize, GM1, did not influence arthropod abundance or diversity in the field (Table 1).

### 2.3. Effects of Maize Type, Year, and Sampling Time on the Community Composition of Arthropods

#### 2.3.1. Time-Dependent Effects of GM1 on Arthropod Community

**Figure 2:**
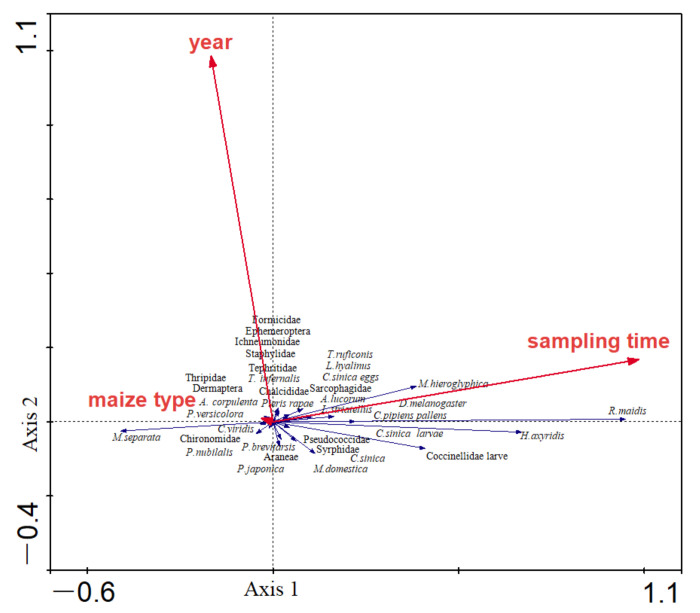
RDA for the composition of arthropod community in the whole plant inspection method, and the relationship of the composition of arthropod community and environmental factors. The red arrows represent variable factors, and the blue arrows represent arthropod species collected in the fields. The length of the blue arrows indicates the magnitude of the effect of environmental factors on arthropods. The cosine of the angle between the red and the blue arrows represents the correlation between variable factors and arthropods.

The effects of maize type, year, and sampling time on the composition of arthropod community were examined using redundancy analysis (RDA). With the whole-plant inspection method, maize type, year, and sampling time explained 36% of the total variation in the composition of the arthropod community. RDA also showed that both sampling time (*p* = 0.0020, *F* = 60.90, and 999 Monte Carlo permutations) and year (*p* = 0.0120, *F* = 3.01, and 999 Monte Carlo permutations) were significantly correlated with the composition of the arthropod community, whereas maize type (*p* = 0.9440, *F* = 0.44, and 999 Monte Carlo permutations) was not correlated with it (Table 2, Figure 2).

**Figure 3:**
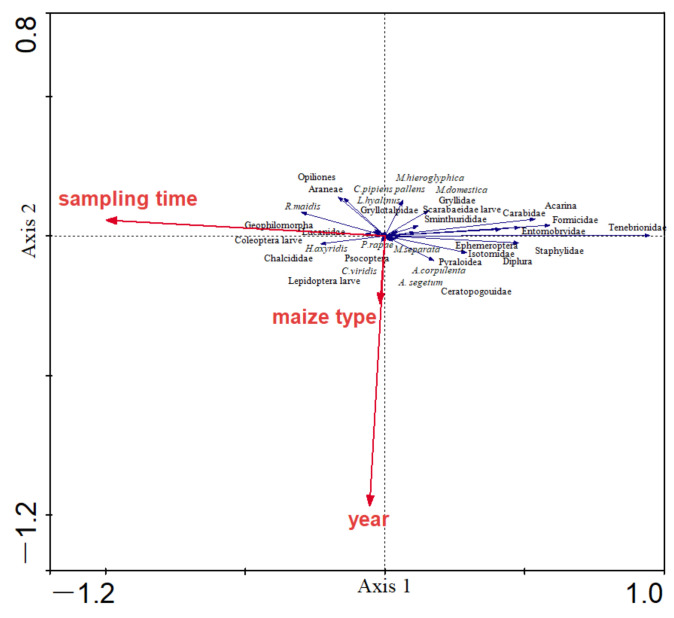
RDA for the composition of arthropod community in the pitfall trap method, and the relationship of the composition of arthropod community and environmental factors. The red arrows represent variable factors, and the blue arrows represent arthropod species collected in the fields. The length of the blue arrows indicates the magnitude of the effect of environmental factors on arthropods. The cosine of the angle between the red and the blue arrows represents the correlation between variable factors and arthropods.

Using the pitfall trap method, RDA revealed that maize type, year, and sampling time, together explained 37% of the variation in the composition of arthropod community. Moreover, RDA showed that the composition of arthropod community was significantly correlated with sampling time (*p* = 0.0020, *F* = 38.50, and 999 Monte Carlo permutations), whereas it was not influenced by maize type (*p* = 0.7660, *F* = 0.62, and 999 Monte Carlo permutations) or year (*p* = 0.1180, *F* = 1.49, and 999 Monte Carlo permutations) (Table 3 and Figure 3).

**Figure 4:**
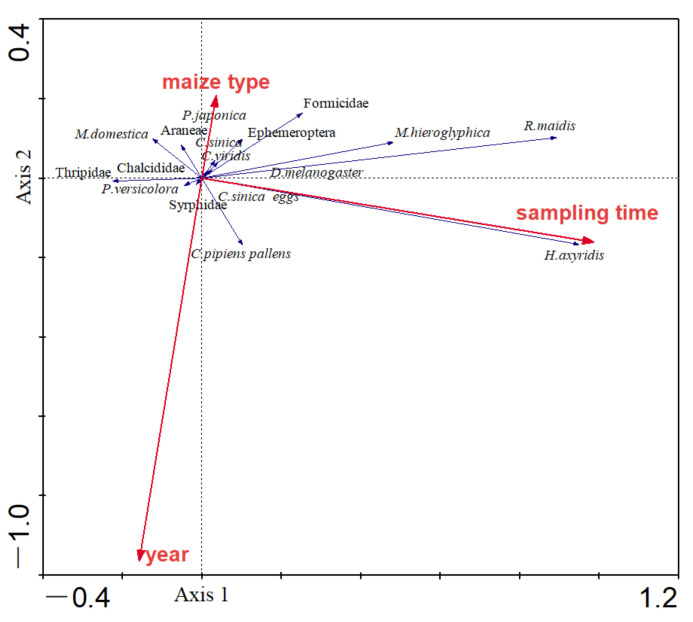
RDA for the composition of arthropod community in the suction sampler method, and the relationship of the composition of arthropod community and environmental factors. The red arrows represent variable factors, and the blue arrows represent arthropod species collected in the fields. The length of the blue arrows indicates the magnitude of the effect of environmental factors on arthropods. The cosine of the angle between the red and the blue arrows represents the correlation between variable factors and arthropods.

Using the suction sampler method, RDA indicated that maize type, year, and sampling time together explained 33% of the variation of composition of arthropod community. Furthermore, RDA showed that both sampling time (*p* = 0.0020, *F* = 23.71, and 999 Monte Carlo permutations) and year (*p* = 0.0480, *F* = 2.10, and 999 Monte Carlo permutations) significantly correlated with arthropod community composition but not with maize type (*p* = 0.8880, *F* = 0.50, and 999 Monte Carlo permutations) (Table 4, Figure 4).

#### 2.3.2. Similarity of Arthropod Communities in Transgenic and Non-Transgenic Maize Fields

**Figure 5:**
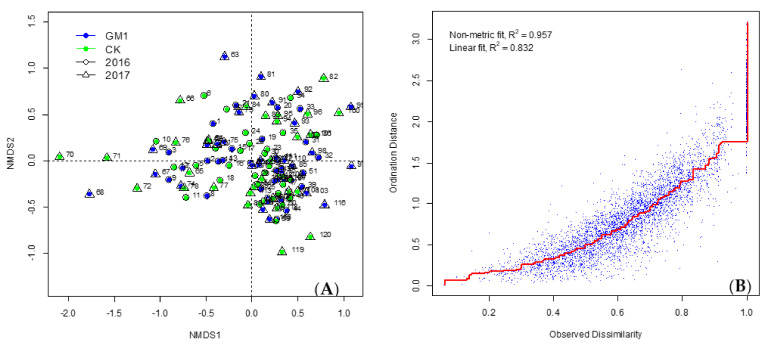
nMDS plot of community structures of arthropods from GM1 and CK in each sampling time in 2016 and 2017 using the whole plant inspection method. (**A**): Blue indicates transgenic maize, and green indicates non-transgenic maize. The circles with the numbers 1 to 60 indicate the sampling points in 2016. Numbers 1–3, 7–9, 13–15, 19–21, 25–27, 31–33, 37–39, 43–45, 49–51, 55–57: transgenic maize from 1st to 10th stage respectively; while 4–6, 10–12, 16–18, 22–24, 28–30, 34–36, 40–42, 46–48, 52–54, 58–60: non-transgenic maize from 1st to 10th stage respectively. The triangles with the numbers 61 to 120 indicate the sampling points in 2017. Numbers 61–63, 67–69, 73–75, 79–81, 85–87, 91–93, 97–99, 103–105, 109–111, 115–117: transgenic maize from 1st to 10th stage respectively; while 64–66, 70–72, 76–78, 82–84, 88–90, 94–96, 100–102, 106–108, 112–114, 118–120: non-transgenic maize from 1st to 10th stage respectively. (**B**): Shepard stress plot.

**Figure 6:**
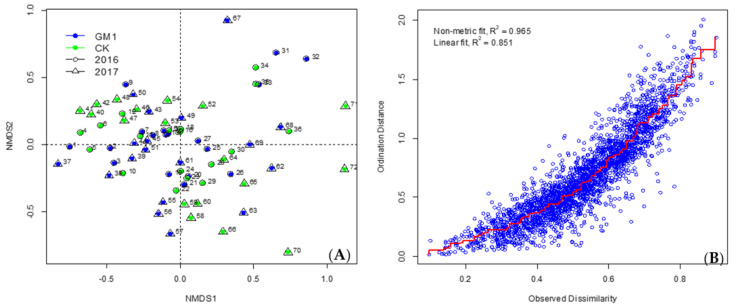
nMDS plot of community structures of arthropods from GM1 and CK in each sampling time in 2016 and 2017 using the pitfall trap method. (**A**): Blue indicates transgenic maize, and green indicates non-transgenic maize. The circles with the numbers 1 to 36 indicate the sampling points in 2016. Numbers 1–3, 7–9, 13–15, 19–21, 25–27, 31–33: transgenic maize from 1st to 6th stage respectively; while 4–6, 10–12, 16–18, 22–24, 28–30, 34–36: non-transgenic maize from 1st to 6th stage respectively. The triangles with the numbers 37 to 72 indicate the sampling points in 2017. Numbers 37–39, 43–45, 49–51, 55–57, 61–63, 67–69: transgenic maize from 1st to 6th stage respectively, while 40–42, 46–48, 52–54, 58–60, 64–66, 70–72: non-transgenic maize from 1st to 6th stage respectively. (**B**): Shepard stress plot.

**Figure 7:**
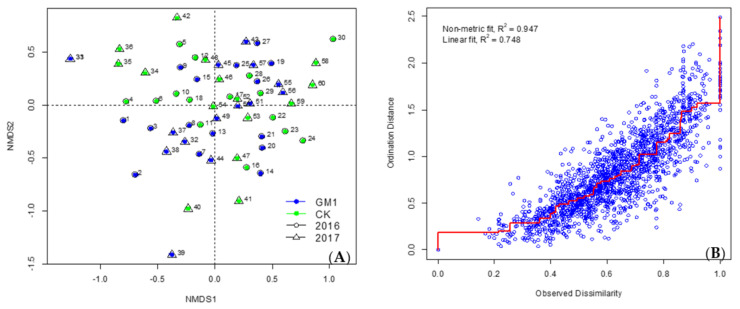
nMDS plot of community structures of arthropods from GM1 and CK in each sampling time in 2016 and 2017 by the suction sampler method. (**A**): Blue indicates transgenic maize, and green indicates non-transgenic maize. The circles with the numbers 1 to 30 indicate the sampling points in 2016. Numbers 1–3, 7–9, 13–15, 19–21, 25–27: transgenic maize from 1st to 5th stage respectively; while 4–6, 10–12, 16–18, 22–24, 28–30: non-transgenic maize from 1st to 5th stage respectively. The triangles with the numbers 31 to 60 indicate the sampling points in 2017. Numbers 31–33, 37–39, 43–45, 49–51, 55–57: transgenic maize from 1st to 5th stage respectively; while 34–36, 40–42, 46–48, 52–54, 58–60: non-transgenic maize from 1st to 5th stage respectively). (**B**): Shepard stress plot.

The arthropod community structures in transgenic and non-transgenic maize fields were further explored using non-metric multidimensional scaling analysis (nMDS). The distance between the two sampling points was estimated using the Bray–Curtis dissimilarity index. Differences in the composition of arthropod community among all animal samples were visualized in the nMDS plot. The samples collected in the nMDS plot were separated by sampling time but not by maize type or year (Figure 5, Figure 6 and Figure 7), which was confirmed by a more detailed analysis of similarity (ANOSIM). A significant correlation was detected between arthropod community composition and sampling time, but arthropods were not affected by maize type or year (Table 5).

## 3. Discussion

Biodiversity of arthropod communities is an important evaluation index for the safety of genetically modified crops. We present the analysis of the arthropod communities affected by the cultivation of the genetically modified drought-resistant maize cultivar, GM1. We provide basic safety data for the risk assessment associated to the commercialization of drought-resistant maize, GM1.

Although GM crops have significantly improved crop quality and resistance, they have also sparked intense debate regarding their safety [31]. With the increase in commercial applications and the expansion of the planting area of GM crops, their biosafety to arthropods has become a major concern. To date, studies on genetically modified insect- and herbicide-resistant maize have shown that maize type has no significant effect on arthropod diversity [32,33,34,35,36,37,38]. Moreover, it has been demonstrated that maize type and year do not have significant impacts on biodiversity [39,40,41,42]. Genetically modified wheat, TB4, has no significant effects on the content of available nutrients, enzyme activities, or diversity of the microbial community in the soil rhizosphere [25]. Similarly, drought-tolerant wheat (*Triticum aestivum* L.) and genetically modified dehydration-responsive element-binding 3 (DREB3) drought-resistant soybeans have low potential weediness [26,27]. A significant effect on the number of bacteria, actinomycetes, fungi, *Trichoderma*, or *Azotobacter* in the rhizosphere of transgenic drought resistant soybean was not observed [28]. In our 2-year field trial conducted under natural conditions, we found that genetically modified drought-resistant maize, GM1, had no significant effect on arthropod biodiversity. This result is consistent with previous studies showing that genetically modified maize did not affect arthropod biodiversity in the field.

In this study, the effects of genetically modified maize showed that sampling time and year were more related to the diversity and richness of arthropods than maize type. The results of Guo and Fan support this view [17,18,40]. Furthermore, RDA showed that sampling time and year were the leading causes of variation in the arthropod community composition, which may be explained as follows: (1) sampling times differ with weather conditions, (2) sampling years differ in climatic conditions, and (3) arthropods are influenced by climate conditions in a complex manner. The multivariate permutation test for analyzing the effects of maize type, year, and sampling time showed that sampling time had a close relationship with Bray–Curtis distances but not maize type or year. Arthropod diversity and composition were also affected by environmental factors, such as climate, humidity, temperature, and light [41], which may explain why sampling time was the main reason for variation.

Analysis of five parameters, including Shannon–Wiener, Pielou’s, Simpson’s indexes, number of species, and the total number of individuals, showed that GM1 had a negligible effect on arthropod abundance and diversity. Furthermore, RDA and nMDS of the variable factors in the three investigation methods indicated that the arthropod community composition was not associated with maize type. Moreover, the three arthropod investigation methods used in this study complemented each other to provide a more comprehensive collection of arthropods [43,44,45]. However, using these three methods simultaneously is an intensive and complex operation; hence, we are trying to simplify the sampling method without compromising a comprehensive sample collection.

This field study evaluated the environmental safety of drought-resistant genetically modified maize, GM1, in Jilin in 2016 and 2017, when annual precipitation was normal (drought). Results revealed that the maize type had no impact on the diversity, abundance, or composition of the community of arthropods. Therefore, we concluded that planting drought-resistant genetically modified maize GM1 in Jilin Province will not adversely affect the environment.

## 4. Materials and Methods

### 4.1. Experimental Materials

Genetically modified DREB maize (GM1) and its non-transformed counterpart (CK) were used in this study.

### 4.2. Experimental Field

Safety assessment trials were conducted in 2016 and 2017 at the Jilin Academy of Agricultural Sciences in Gongzhuling, Jilin Province, China (43°30′ N, 124°49′ E).

Jilin Province is a vital spring maize production region in China; however, Jilin is a rainfed agricultural area, in which various degrees of drought occur annually [46]. In the 2 years of study, precipitation was normal, which was 772.5 and 546.9 mm in 2016 and 2017, respectively, and it mostly occurred from May to September (84.3% in 2016 and 94.5% in 2017). Meteorological data were obtained from the Gongzhuling Meteorological Observatory in Jilin.

### 4.3. Experimental Design

Maize seeds were planted on 5 May in 2016 and 2017. Three replicate plots for each cultivar (GM1 and Chang 7-2 (CK)) were established in a completely randomized design [17,18,39,40,47]. According to the National Standards of the People’s Republic of China, Ministry of Agriculture Announcement No. 2122-10.4-2014, the following experimental set up was established: Each plot was 10 m wide and 15 m long and contained 25 rows with 60 cm spacing. There were 40 plants in each row, 25 cm apart. No pesticides were applied during the growth period [48]. This study was approved by the Ministry of Agriculture of the People’s Republic of China and by the Genetically Modified Organism Safety Team at the Jilin Academy of Agricultural Sciences, China. The field study did not involve endangered or protected species.

### 4.4. Sample Collection Methods

The Ministry of Agriculture Announcement No. 2122-10.4-2014 requires the use of whole plant inspection, pitfall trap, and suction samplers to evaluate the impact of biodiversity [48]. The whole plant inspection method was more flexible and simpler, the suction sampler was very effective in catching small and flying arthropods, and the pitfall trap method could continuously collect ground-dwelling arthropods in the daytime and at night [43,44,45]. The use of these methods provides a more comprehensive collection of arthropods.

#### 4.4.1. Whole Plant Inspection Method

Arthropods were investigated every 7 d (depending on the weather) from day 10 after the final plant thinning to maturity. A five-spot sampling method was used to investigate the arthropods in each plot: one was located at the center of the plot, and the other four were located at the middle of the lines that connected the center of the plot and the plot corners. At each sampling spot, we randomly and gently turned over five maize plants one by one and quickly counted the visible arthropods on the plant surface and on the ground within 1 m^2^ around the plant. During the investigation, the number of active insects and spiders were counted.

#### 4.4.2. Pitfall Trap Method

Arthropods were investigated every 10 d (depending on the weather) from day 10 after final thinning to maturity. A five-spot sampling method was used. Three pitfall traps with a spacing of 0.5 m were established at each sampling spot in every plot. For each trap, a plastic cup (15 cm in diameter × 10 cm in depth) was buried in the soil, with the upper rim of the cup level with the ground. Each cup was then placed in 5% detergent diluent (no more than 1/3 of the cup volume). Traps were exposed to the field for approximately 24 h, after which they were removed, and the trapped arthropods were collected and stored in 75% ethanol for species and number identification.

#### 4.4.3. Suction Sampler Method

The first investigation was conducted 15 days after final thinning of maize, and then it was repeated at the mid-whorl and late whorl stages, at the peak of the silking stage, and the late grain-filling stage. Arthropods were investigated five times in total using this method. A five-spot sampling method was used again in this case. At each sampling site, five maize plants were randomly selected, and the arthropods on the whole maize plant and on the ground within 1 m^2^ of the plant were extracted with a suction sampler (John W. Hock Co. Gainesville, FL, USA).

The unknown species sampled by the above methods were collected and stored in a 5 mL plastic tube containing 75% alcohol and brought to the laboratory for identification under an insect anatomical lens (Motic, Xiamen City, China).

### 4.5. Statistical Analysis

As a single method cannot comprehensively evaluate the arthropod community, it is necessary to use a combination of methods to obtain a more comprehensive understanding. Therefore, diversity indices (Shannon–Weiner and Simpson’s indexes, and for evenness, Pielou’s index), abundance indices, redundancy analysis (RDA), and nonmetric multidimensional scaling (nMDS) were used in this study.

Arthropod diversity and abundance were analyzed using the Data Processing System (DPS, version 2006 package, China). The Shannon–Wiener index (*H*′), Simpson’s diversity index (*D*), and Pielou’s index (*J*) were calculated as follows:

(1) $$H^{\prime }=-\overset{S}{\underset{i=1}{\sum }}P_{i}\ln\left(P_{i}\right)$$

where *P_i_* is the proportion of individuals belonging to the *i*th taxon in a single plot.

(2) $$D=1-\overset{S}{\underset{i=1}{\sum }}\frac{N_{i}\left(N_{i}-1\right)}{N\left(N-1\right)}$$

where *N_i_* is the individual number in the *i*th taxon in one plot, and *N* is the total number of animals in one plot.

(3) J=H′lnS

where, *S* is the genus number of the collected arthropods in one plot.

In this study, maize type, year, and sampling time were considered as the factors presumably influencing arthropod abundance and diversity. They were all used as fixed factors when analyzing for repeated-measures ANOVA (SPSS 23.0).

According to Guo’s research in 2016 [17,18], this study used RDA, a canonical analysis for studying environmental safety and analyze the relationships among arthropods and the relationships between arthropods and maize type, year, or sampling time, to identify the influencing factors of arthropod community. RDA can be performed using CANOCO 4.5 [41,49]. In addition, nonmetric multidimensional scaling (nMDS) was used to indicate the effects of each influencing factor on arthropod community, which is an indirect ordination method that can reveal the similarity of arthropod samples through metric multidimensional scale analysis using the Bray–Curtis distance between sampling points [50,51]. nMDS was conducted using the Vegan package in R (v.4.0.4; R Development Core Team).

## 5. Conclusions

Maize variety did not influence arthropod abundance, diversity, or community composition over 2 consecutive years of study in the field. Further, RDA indicated that maize variety did not significantly affect arthropod community composition. These results strongly suggest that the cultivation of DREB-GM maize does not affect the arthropod community. However, considering the limitations of time and area of influence inherent to this study, long-term field experimentation in a larger area is necessary to guarantee the environmental and ecological safety of genetically modified crop plants.

## Figures and Tables

**Table 1:** Multi-factor effects of year (2016 and 2017), maize type (GM1 and CK), and sampling time on diversity and abundance of arthropods.

Investigation Method	Factor		Shannon–Weiner’s Index (*H’*)		Simpson’s Diversity Index (*D*)		Pielou’s Evenness Index (*J*)		Number of Species (*S*)		Total Abundance (*N*)		Abundance of Critical Groups			
													*Rhopalosiphum maidis*		Formicidae	
			*F*	*p*	*F*	*p*	*F*	*p*	*F*	*p*	*F*	*p*	*F*	*p*	*F*	*p*
**Whole plant inspection method**	Year		18.823	0.002 **	61.658	0.000 ***	80.475	0.000 ***	3.964	0.082	0.212	0.657	0	1	2.462	0.155
	Maize type		0.064	0.807	0.038	0.85	0.823	0.391	0.367	0.561	1.156	0.314	2.709	0.138	0.427	0.532
	Sampling time		19.56	0.000 ***	38.71	0.000 ***	84.41	0.000 ***	21.41	0.000 ***	244.91	0.000 ***	306.456	0.000 ***	10.178	0.000 ***
	Maize type with Year		0.202	0.665	0.035	0.857	0.274	0.615	0.187	0.677	0.001	0.979	0.142	0.717	1.094	0.326
	Year with Sampling time		15.32	0.000 ***	38.31	0.000 ***	54.47	0.000 ***	2.93	0.041 *	20.33	0.000 ***	53.152	0.000 ***	3.427	0.032 *
	Maize type with Sampling time		0.30	0.863	0.10	0.964	0.32	0.868	0.70	0.588	1.09	0.38	1.881	0.179	0.98	0.42
	Maize type with Year and Sampling time		0.21	0.921	0.20	0.905	0.38	0.824	0.35	0.828	2.32	0.07	2.158	0.141	0.344	0.8
	Mean ± SD (GM1)		2.312 ± 0.171		0.733 ± 0.028		0.756 ± 0.019		8.866 ± 1.063		37.484 ± 3.085		12.967 ± 1.026		1.467 ± 0.503	
	Mean ± SD (CK)		2.288 ± 0.170		0.730 ± 0.033		0.765 ± 0.022		8.517 ± 0.917		35.499 ± 2.451		11.800 ± 1.293		1.300 ± 0.626	
**Pitfall trap method**	Year		23.087	0.001 ***	14.533	0.005 **	12.843	0.007 **	5.911	0.041 *	3.275	0.108	0.181	0.682	1.852	0.211
	Maize type		2.756	0.135	2.831	0.131	0.024	0.882	4.034	0.079	0.078	0.787	0.647	0.445	4.605	0.064
	Sampling time		60.849	0.000 ***	85.218	0.000 ***	163.107	0.000 ***	23.25	0.000 ***	213.789	0.000 ***	46.295	0.000 ***	31.194	0.000 ***
	Maize type with Year		1.695	0.229	1.798	0.217	0.114	0.745	3.229	0.11	1.719	0.226	4.942	0.057	4.274	0.073
	Year with Sampling time		0.6	0.7	0.884	0.436	1.05	0.373	0.352	0.878	7.037	0.000 ***	8.438	0.006 **	1.332	0.292
	Maize type with Sampling time		1.376	0.254	3.29	0.061	1.51	0.251	1.02	0.420	1.738	0.148	0.659	0.506	1.52	0.25
	Maize type with Year and Sampling time		1.339	0.268	2.37	0.123	1.69	0.216	1.48	0.219	0.902	0.489	1.971	0.181	0.743	0.483
	Mean ± SD (GM1)		2.831 ± 0.148		0.799 ± 0.027		0.769 ± 0.023		13.389 ± 0.977		99.584 ± 8.970		4.000 ± 0.920		23.028 ± 5.218	
	Mean ± SD (CK)		2.733 ± 0.101		0.780 ± 0.020		0.767 ± 0.021		12.333 ± 0.802		101.390 ± 8.549		3.528 ± 0.721		18.472 ± 2.981	
**Suction sampler method**	Year		0.578	0.469	5.416	0.048 *	12.626	0.007 **	1.84	0.212	5.788	0.043 *	1.903	0.205	8.696	0.018 *
	Maize type		0.05	0.829	2.476	0.154	2.288	0.169	0.115	0.743	0.027	0.874	0.004	0.954	4.261	0.073
	Sampling time		9.825	0.000 ***	22.19	0.000 ***	41.108	0.000 ***	14.973	0.000 ***	104.592	0.000 ***	49.17	0.000 ***	17.171	0.000 ***
	Maize type with Year		0.078	0.787	0.319	0.587	2.917	0.126	0.013	0.913	0.197	0.669	0.291	0.604	4.261	0.073
	Year with Sampling time		1.122	0.364	0.451	0.653	2.568	0.057	0.50	0.739	3.107	0.029 *	5.148	0.003 **	7.805	0.000 ***
	Maize type with Sampling time		0.816	0.524	0.73	0.501	1.31	0.286	0.42	0.794	1.536	0.215	1.36	0.27	2.341	0.076
	Maize type with Year and Sampling time		0.412	0.799	0.62	0.555	1.71	0.173	0.68	0.614	0.483	0.748	0.556	0.696	0.39	0.814
	Mean ± SD (GM1)		2.277 ± 0.227		0.860 ± 0.020		0.903 ± 0.017		6.300 ± 0.937		14.333 ± 2.014		3.567 ± 0.699		0.900 ± 0.307	
	Mean ± SD (CK)		2.244 ± 0.212		0.831 ± 0.040		0.888 ± 0.024		6.100 ± 0.806		14.567 ± 1.665		3.533 ± 0.911		1.367 ± 0.482	

The values in bold font are statistically significant (* *p* < 0.05; ** *p* < 0.01; *** *p* < 0.001).

**Table 2:** Monte Carlo permutation test for variable factors in the whole plant inspection method.

Variable Factor	Proportion of Variance Explained (%)	*p*	*F*
Sampling time	34	0.0020 **	60.90
Year	2	0.0120 *	3.01
Maize type	0	0.9440	0.44
Total	36		

The values in bold font are statistically significant (* *p* < 0.05; ** *p* < 0.01).

**Table 3:** Monte Carlo permutation test for the variable factors in the pitfall trap method.

Variable Factor	Proportion of Variance Explained (%)	*p*	*F*
Sampling time	35	0.0020 **	38.50
Year	1	0.1180	1.49
Maize type	1	0.7660	0.62
Total	37		

The values in bold font are statistically significant (** *p* < 0.01).

**Table 4:** Monte Carlo permutation test of the suction sampler method.

Variable Factor	Proportion of Variance Explained (%)	*p*	*F*
Sampling time	29	0.0020 **	23.71
Year	3	0.0480 *	2.10
Maize type	1	0.8880	0.50
Total	33		

The values in bold font are statistically significant (* *p* < 0.05; ** *p* < 0.01).

**Table 5:** Effects of maize type (GM1 and CK), year, and sampling time on the community structure of arthropods (nMDS structure) in 2016 and 2017.

Correlation with nMDS Structure	Whole Plant Inspection Method		Pitfall Trap Method		Suction Sampler Method	
	R^2^	*p*	R^2^	*p*	R^2^	*p*
Sampling time	0.80	0.001 ***	0.67	0.001 ***	0.49	0.001 ***
Year	0.00	0.07	0.00	0.26	0.00	0.112
Maize type	0.00	0.994	0.00	0.897	0.00	0.986

The values in bold font are statistically significant (*** *p* < 0.001).

## Data Availability

The data presented in this study are available on request from the corresponding author. The data are not publicly available due to privacy.

## References

[1] Li B., Wang Z.C., Sun Z.G., Chen Y., Yang F. (2005). Resources and sustainable resource exploitation of salinized land in China. Agric. Res. Arid. Areas.

[2] Ji J.H., Li Y.Y., Liu S.Q., Tong Y.X. (2015). Effect of Plastic Film Mulch and Drip Irrigation under Plastic Film Mulch on Growth and Development of Maize and Water use Efficiency. Water Sav. Irrig..

[3] Yu G.R., Zhang W., Du W.P., Song J., Chen Q., Xu L.Y. (2016). Transgenic Drought-resistant Gene IrrE Maize Material Obtaining and Resistance Identification. Southwest China J. Agric. Sci..

[4] Liu B., Zeng Q., Yan F.M., Xu H.G., Xu C.R. (2005). Effects of transgenic plants on soil microorganisms. Plant Soil.

[5] Truter J., Hamburg H.V., Berg J.V.D. (2014). Comparative diversity of arthropods on Bt maize and non-Bt maize in two different cropping systems in South Africa. Environ. Entomol..

[6] Yu H.L., Li Y.H., Li X.J., Wu K.M. (2014). Arthropod Abundance and Diversity in Transgenic Bt Soybean. Environ. Entomol..

[7] Bal H.K., Dhawan A.K. (2010). Impact of intercropping on arthropod diversity in Bt and non-Bt cotton. J. Insect Sci..

[8] Bal H.K., Dhawan A.K. (2009). Impact of transgenic cotton on diversity of non-target arthropod communities in cotton agro-ecosystem. J. Insect Sci..

[9] Romeis J., Hellmich R.L., Candolfi M.P., Carstens K., Schrijver A.D., Gatehouse A.M.R., Herman R.A., Huesing J.E., Mclean M.A., Raybould A. (2011). Recommendations for the design of laboratory studies on non-target arthropods for risk assessment of genetically engineered plants. Transgenic Res..

[10] Romeis J., Raybould A., Bigler F., Candolfi M.P., Hellmich R.L., Huesing J.E., Shelton A.M. (2013). Deriving criteria to select arthropod species for laboratory tests to assess the ecological risks from cultivating arthropod-resistant genetically engineered crops. Chemosphere.

[11] Romeis J., Meissle M., Alvarez-Alfageme F., Bigler F., Bohan D.A., Devos Y., Malone L.A., Pons X., Rauschen S. (2014). Potential use of an arthropod database to support the non-target risk assessment and monitoring of transgenic plants. Transgenic Res..

[12] Szénási A., Pálinkás Z., Zalai M., Schmitz O.J., Adalbert B. (2014). Short-term effects of different genetically modified maize varieties on arthropod food web properties: An experimental field assessment. Sci. Rep..

[13] Hu H.H., Xiong L.Z. (2014). Genetic Engineering and Breeding of Drought-Resistant Crops. Annu. Rev. Plant Biol..

[14] Kang Q., Vahl C.I. (2014). Statistical Analysis in the Safety Evaluation of Genetically-Modified Crops: Equivalence Tests. Crop Sci..

[15] Zhang M.D., Sun L., Xiong Q.F. (2015). The Safety Evaluation of the Genetically Modified Crops. Hubei Agric. Sci..

[16] Ren Z.T., Shen W.J., Liu B., Xue K. (2017). Effects of Transgenic Maize on Biodiversity of Arthropod Communities in the Fields. Sci. Agric. Sin..

[17] Guo J.F., He K.L., Bai S.X., Zhang T.T., Liu Y.J., Wang F.X., Wang Z.Y. (2016). Effects of transgenic cry1Ie maize on non-lepidopteran pest abundance, diversity and community composition. Transgenic Res..

[18] Guo J.F., He K.L., Hellmich R.L., Bai S.X., Zhang T.T., Liu Y.J., Ahmed T., Wang Z.Y. (2016). Field trials to evaluate the effects of transgenic cry1Ie maize on the community characteristics of arthropod natural enemies. Sci. Rep..

[19] Guo Y.Y., Feng Y.J., Ge Y., Tetreau G., Chen X.W., Dong X.H., Shi W.P. (2014). The Cultivation of Bt Corn Producing Cry1Ac Toxins Does Not Adversely Affect Non-Target Arthropods. PLoS ONE.

[20] Habustova O., Dolezal P., Spitzer L., Svobodova Z., Hussein H., Sehnal F. (2014). Impact of Cry1Ab toxin expression on the non-target insects dwelling on maize plants. J. Appl. Entomol..

[21] Rauschen S., Schultheis E., Hunfeld H., Schaarschmidt F., Schuphan I., Eber S. (2010). Diabrotica-resistant, Bt-maize, DKc5143 event MON88017 has no impact on the field densities of the leafhopper, Zyginidia scutellaris. Environ. Biosaf. Res..

[22] Xu Z.S., Chen M., Li L.C., Ma Y.Z. (2008). Functions of the ERF transcription factor family in plants. Botany.

[23] Xu Z.S., Chen M., Li L.C., Ma Y.Z. (2011). Functions and application of the AP2/ERF transcription factor family in crop improvement. J. Integr. Plant Biol..

[24] Xu Z.S., Ni Z.Y., Liu L., Nie L.N., Li L.C., Chen M., Ma Y.Z. (2008). Characterization of the TaAIDFa gene encoding a CRT/DRE-binding factor responsive to drought, high-salt, and cold stress in wheat. Mol. Genet. Genom..

[25] Jiang Q.Y., Li X.H., Hu Z., Ma Y.Z., Zhang H., Xu Z.S. (2015). Safety Assessment of Weediness of Transgenic Drought-Tolerant Wheat (*Triticum aestivum* L.). Sci. Agric. Sin..

[26] Yu M.Z., Dai R.Y., Wu J.R., Xu J.H., Du J., Chen S.L., Shi J.R. (2013). Analysis of available nutrient, enzyme activities and microorganism community diversity in rhizospheric soil of TaDREB4 transgenic wheat with drought resistance. Jiangsu J. Agric. Sci..

[27] Wang J.Y., Ding W., Muhammad S.K., Cheng Z., Dai H.Y. (2018). Survival competition between transgenic drought-resistant soybean and weeds with different carbon metabolism pathways under drought stress. Jiangsu Agric. Sci..

[28] Cao Y., Ding W., Li X.H., Ma Y.Z., Wang Z.H., Li W.B. (2011). Effect of transgenic drought resistant soybean on soil microbial community and beneficial microorganism. J. Northeast Agric. Univ..

[29] Xing S., Yao Y., Lei X., Hu X., Yang L. (2014). Advances of Drought-resistant Genes and Drought-resistant Transgenic of Main Crops of Gramineae Plants. Chin. Agric. Sci. Bull..

[30] Shirai Y. (2004). Influence of transgenic insecticidal crops on non-target arthropods: A review. Jpn. J. Ecol..

[31] Cui K., Shoemaker S.P. (2018). Public perception of genetically modified (GM) food: A Nationwide Chinese consumer study. Sci. Food.

[32] Li Y.H., Zhang X.J., Chen X.P., Romeis J., Yin X.M., Peng Y.F. (2015). Consumption of Bt rice pollen containing Cry1C or Cry2A does not pose a risk to Propylea japonica (Thunberg) (Coleoptera: Coccinellidae). Sci. Rep..

[33] Wang Y.Y., Li Y.H., Romeis J., Chen X.P., Zhang J., Chen H.Y., Peng Y.F. (2012). Consumption of Bt rice pollen expressing Cry2Aa does not cause adverse effects on adult Chrysoperla sinica Tjeder (Neuroptera: Chrysopidae). Biol. Control.

[34] Yang Y., Liu Y., Cao F.Q., Chen X.P., Chen L.S., Romeis J., Li Y.H., Peng Y.F. (2014). Consumption of Bt Rice Pollen Containing Cry1C or Cry2A Protein Poses a Low to Negligible Risk to the Silkworm Bombyx mori (Lepidoptera: Bombyxidae). PLoS ONE.

[35] Ahmad A., Wilde G.E., Zhu K.Y. (2005). Detectability of Coleopteran-specific Cry3Bb1 Protein in Soil and Its Effect on Nontarget Surface and Below-Ground Arthropods. Environ. Entomol..

[36] Li X.G., Liu B., Desneux N. (2013). A 2-Year Field Study Shows Little Evidence That the Long-Term Planting of Transgenic Insect-Resistant Cotton Affects the Community Structure of Soil Nematodes. PLoS ONE.

[37] Romeis J., Bartsch D., Bigler F., Candolfi M.P., Gielkens M.M.C., Hartley S.E., Hellmich R.L., Huesing J.E., Jepson P.C., Layton R. (2008). Assessment of risk of insect-resistant transgenic crops to nontarget arthropods. Nat. Biotechnol..

[38] Li Y.H., Romeis J., Wu K.M., Peng Y.F. (2014). Tier-1 assays for assessing the toxicity of insecticidal proteins produced by genetically engineered plants to non-target arthropods. Insect Sci..

[39] Wang B.F., Wu F.C., Yin J.Q., Jiang Z.L., Song X.Y., Reddy G.V.P. (2021). Use of taxonomic and trait-based approaches to evaluate the effect of bt maize expressing cry1ie protein on non-target collembola: A case study in northeast china. Insects.

[40] Fan C.M., Wu F.C., Dong J.Y., Wang B.F., Yin J.Q., Song X.Y. (2019). No impact of transgenic cry1Ie maize on the diversity, abundance and composition of soil fauna in a 2-year field trial. Sci. Rep..

[41] Song X.Y., Chang L., Reddy G.V.P., Zhang L., Fan C.M., Wang B.F. (2018). Use of taxonomic and trait-based approaches to evaluate the effects of transgenic cry1ac corn on the community characteristics of soil collembola. Environ. Entomol..

[42] Carpenter J.E. (2015). Impact of GM crops on biodiversity. Communities of ground-dwelling arthropods in conventional and transgenic maize: Background data for the post-market environmental monitoring. J. Appl. Entomol..

[43] Bogya S., Markó V. (1999). Effect of pest management systems on ground-dwelling spider assemblages in an apple orchard in Hungary. Agric. Ecosyst. Environ..

[44] Liu J., Chen J. (2015). Effects of transgenetic Bt cotton on ground-dwelling spider assemblages by pitfall traps. J. Plant Prot..

[45] Wang Y., Chen J., Xiao D.H., Ma F.G., Hua H.X. (2016). Assessing the efficacy of different sampling methods for arthropods in rice field. J. Environ. Entomol..

[46] Mu J., Qiu M.J., Gu Y., Ren J.Q., Liu Y. (2018). Applicability of five drought indices for agricultural drought evaluation in Jilin Province, China. Chin. J. Appl. Ecol..

[47] Liang J.G., Meng F., Sun S., Wu C.X., Wu H.Y., Zhang M.R., Zhang H.F., Zheng X.B., Song X.Y., Zhang Z.G. (2015). Community structure of arbuscular mycorrhizal fungi in rhizospheric soil of a transgenic high-methionine soybean and a near isogenic variety. PLoS ONE.

[48] Ministry of Agriculture of the PRC (2003). NY/T 720.3-2003 Environmental impact testing of genetically modified maize-Part 3:testing the effects on biodiversity.

[49] Braak C.J.F.T., Smilauer P. (2002). CANOCO reference manual andCanoDraw for Windows user’s guide: Software for canonical communityordination (version 4.5).

[50] Jackson M.M., Turner M.G., Pearson S.M., Ives A.R. (2012). Seeing the forest and the trees: Multilevel models reveal both species and community patterns. Ecosphere.

[51] Clarke K.R. (2010). Non-parametric multivariate analyses of changes in community structure. Aust. J. Ecol..

